# Obstetrics and Gynecology

**Published:** 1896-04

**Authors:** 


### SELECTIONS AND ABSTRACTS.

### OBSTETRICS AND GYNECOLOGY.

EDITED BY DR. VIRGIL O. HARDON.

### THE AFTER-TREATMENT OF CELIOTOMY.

REICHEL (*Archiv für klinische Chirurgie*, Band L, Heft 2) considers the question as to whether it is advisable after the operation of celiotomy to stimulate or depress peristalsis, and, further, what influence treatment has upon intestinal paresis or ileus. He believes that, regardless of the fact that the operation may be aseptic, there may be a migration of bacteria, that these bacteria may be distributed over the entire peritoneum and may possibly be quickly absorbed and destroyed, but that on the other hand, diffuse or circumscribed purulent peritonitis may result. Also, that if this purulent peritonitis is isolated the prognosis is always better. Granted that this is true, he concludes that in cases of intestinal resection and those celiotomies in which a portion of infected material is allowed to remain in the abdominal cavity, that the arrest of peristalsis is not advisable; that the administration of opium does harm. He advises that from the day of operation peristalsis should be slightly stimulated by the administration of small doses of saline water and the bowels should be opened on the second or third day. Should the symptoms of subacute intestinal paresis or ileus appear, probably due to intestinal adhesions, no food and only a small amount of water should be given; also that the stomach should be repeatedly washed out and opium given in suppository. Saline cathartics are now contraindicated. If the patient's condition improves, an enema of water containing glycerine should be administered. If, after twenty-four hours, or at the latest forty-eight hours, the patient's condition has not improved, the abdomen should be reopened. Where the trouble is thought to be due to mechanical occlusion of the intestine, as where there is advanced peritonitis, purgation is strongly contraindicated. Reichel believes that simple mechanical occlusion is rare; that the greater number of cases are due to paresis of the intestinal muscle wall where the intestine is bent upon itself or looped; that this paresis and the stenosis is increased and the bowel distended when artificial peristalsis is induced. Therefore this condition is best treated by keeping the intestinal tract at rest. The abdomen should be reopened where there is sepsis and where the symptoms point towards mechanical obstruction.—*Univ. Med. Magazine.*

### NON NOCERE IN OBSTETRICS.

Dr. Julius Rosenberg, in *Medico-Surgical Bulletin,* says:

In obstetrics the axiom, *non nocere,* is of special significance. The birth of a child is a physiological act, yet it is often changed into a pathological condition as a result of ill-directed zeal on the part of the medical attendant. I hold that puerperal infection is an absolutely avoidable complication of childbirth, no matter what the surroundings. Before 1847 the mortality from puerperal infection in the Vienna General Hospital was 10 per cent. Then Semmelweis restricted the number of examinations, and required the washing of the hands in chlorine water. At the end of two years of this practice the mortality had been reduced to 1½ per cent. This fact alone is exceedingly instructive. It cannot be denied that the vagina of every woman contains micro-organisms, but these are not pathogenic. It has been demonstrated that not only is the vaginal secretion in normal pregnancy free from pathogenic germs, but it has been shown that the vaginal secretions possess a germicidal power. It has also been shown that syringing the vagina with an antiseptic solution destroys this germicidal power; hence the preliminary antiseptic douche is actually harmful. Leopold and Goldberg have published the statistics of several thousand cases of labor treated both with and without antiseptic douches, and their best results were obtained by omitting the preliminary douche.

Personally, I never employ vaginal douches except under strict indications, and every one of a considerable number of cases during the past few years has recovered. I have just seen a case of quick normal labor, in which the physician ordered carbolic acid douches three times a day, with the result that the woman now has a pulse of 140 and a temperature of 104, and all the symptoms of acute sepsis. It is true that the surroundings were not favorable; but I am sure that there were no indications for the douching, and I think that it was directly responsible in this case for the sepsis.

---

### Significance of Vaginal Discharges.

A leucorrhea, inodorous or of mild odor, persisting during the climacteric, accompanied by increasing hemorrhage, is suspicious, and demands investigation.

A leucorrhea profuse, of peculiarly fetid odor, grumous, excoriating, appearing early or late during the climacteric, with profuse hemorrhage, is reasonable evidence of cancer of the cervix.

A leucorrhea moderate in amount, ill-smelling (the peculiarly fetid odor of cancer of the cervix being absent), accompanied by hemorrhage, suggests cancer of the corpus uteri.

A leucorrheal discharge with hemorrhage, containing material like the washings of meat, is said to indicate sarcoma.

A watery discharge, as a rule, occurring during menstruation, odorless or of little odor, persisting, accompanied by profuse hemorrhage, indicates fibroids; with little or no hemorrhage, polypi.

Profuse bloody discharges coming on gradually with declining menstruation, ceasing usually with the menstrual flow, point to fibroids.

Persistent profuse discharges of blood occurring spontaneously, arising from sudden exercise or coition, occurring, as a rule, after the menopause, indicate cancer.—Mish, *Pacific Medical Journal*, November, 1895.

---

Every tumor first noticed in the breast after the thirty-eight years' epoch is, in the great majority of cases, primarily malignant; in the remainder it is certain, sooner or later, to become associated with malignant features in one form or another.—Snow, *Medical Record*.

